# The threat of COVID-19 will influence consumers’ taste compensation

**DOI:** 10.1038/s41598-025-01245-2

**Published:** 2025-05-30

**Authors:** Xin Yan, Xiong Yan, Xiaoying Huang, Linglan Zhao, Ying Liu

**Affiliations:** 1 Institute of Tourism and Cultural Industry, Yunnan University of Finance and Economics, Kunming, China; 2 School of Tourism and Hotel Management, Yunnan University of Finance and Economics, Kunming, China; 3 School of Mathematics Science, Sichuan Normal University (SICNU), Chengdu, 610066 China; 4 School of Business Administration, Faculty of Business Administration, Southwestern University of Finance and Economics, Chengdu, China

**Keywords:** COVID-19, Mortality salience, Taste compensation, Compensative motivation, Self-efficacy, Strong flavored food, Psychology, Health care

## Abstract

This study examined how taste compensation is affected by mortality salience and its mechanisms during the pandemic. It conducted four studies and confirmed that mortality salience can cause consumers to seek stronger taste compensation, and the mediating role of compensation motivation. Additionally, low self-efficacy leads consumers to rely more on external compensation, as the impact of mortality salience upon compensating motivation can be negatively moderated by self-efficacy. This study identified new antecedents of F&B taste that add to the literature on compensatory consumption and pandemic research. It also helps hospitality managers and marketers to better understand consumers’ dietary preferences and modify their business strategies accordingly during and after the COVID-19 outbreak.

## Introduction

The COVID-19 outbreak triggered a global pandemic, which significantly impacted hospitality industry including restaurants. The current approaches to dealing with these unexpected consequences, such as creating a safer dining environment for customers, are limited to maintaining social distance, reducing service contact, and sanitizing regularly to limit pandemic spread^1,2^. Evaluating the impact of social media on online shopping behavior during COVID-19 pandemic: A Bangladeshi consumers’ perspectives^3^. Perceived daily tension and food cravings and consumption: A within-and between-person investigation^4^.

During the pandemic, as the number of new positive cases per day peaked in China in March 2020, the volume of spicy food logistics increased by 168.4% year on year^5^. Similarly, spicy food delivery volume in South Korea increased dramatically from February to September 2020^5^. Therefore, we begin to consider an intriguing question: do people make compensatory consumption to relieve the psychological tension and fear caused by the COVID-19 by increasing their preference for strongly flavored food?

During the pandemic, individuals are exposed to messages about death, which heightens their awareness of mortality and triggers thoughts about their own demise, leading to increased mortality salience. Previous research has found that heightened mortality salience significantly alters consumers’ purchasing preferences. For instance, they tend to favor domestic products over foreign ones, are more inclined to purchase luxury goods, and prefer prosocial and familiar products. Empirical studies suggest that this behavioral shift occurs as individuals engage in symbolic consumption as psychological defense mechanisms to mitigate existential anxiety^4^.

Compensatory consumption refers to compensating for psychological deficiencies or threats through consumption behaviors that use consumption as an alternative way to meet psychological needs^6^. Sensory compensation, as a distinct component of compensatory consumption, can compensate for psychological deficits or threats through high-intensity sensory experience^7^. When confronted with a threat, people who are experiencing negative emotions such as fear or tension will unconsciously relieve their emotions through physical stimulation^7^. For example, when confronted with a threat, people prefer high-saturation colors and high-decibel music^8^. This type of physiologically strong sensory stimulation will manifest in the sense of taste as well.

Taste, as a sensitive and emotional physical sensory experience, is vulnerable to negative psychological threat. When an individual feels threatened, taste, as a sensory experience, can compensate both physically and psychologically, resulting in taste compensation. On the one hand, people will supplement their energy needs by consuming food for physiological compensation^9^, such as eating spicy foods. On the other hand, different taste experiences can provide psychological compensation^10^. For instance, eating cuisine that tastes like home in a distant country would make people feel more secure^11^. Additionally, people eat more sweets when they’re experiencing depression^12^. The pandemic-affected customers will exhibit a different taste preference in dining consumption than they did in the past if they attempt to seek a stronger flavor experience as a form of psychological compensation. In order to better understand consumer preferences and modify their business strategies, restaurants will benefit from examining how consumers’ taste preferences have changed as a result of the pandemic and illuminating this psychological to physiological process.

## Literature review

### Mortality salience and consumption behavior

Mortality salience (MS) refers to individuals’ awareness of their own mortality^13^. The COVID-19 pandemic has significantly impacted various aspects of people’ s lives. The high infection rates, substantial death tolls, and continuous disease warnings and news reports have kept individuals in a state of heightened fear of death, leading to increased mortality salience. This heightened awareness of mortality is one of the most serious psychological threats facing humanity^14^.

According to Terror Management Theory, even if individuals do not consciously pay much attention to death-related information after exposure, the perceived threat from such encounters can still enter the unconscious psychological level of the individual^15^.

This, in turn, encourages people to change their traditional behaviors, such as consumption and social interaction, in response to mortality salience^16^. As a result, mortality salience will not only result in a negative psychological state but will also have impacts on daily behavior^15^. Specifically, when confronted with mortality salience, people experience a range of negative emotions, including fear of death, anxiety, a sense of lossing control, and so on^17^, which can wreak havoc on their mental health^13^. Negative workplace gossip: its impact on customer service performance and moderating roles of trait mindfulness and forgiveness^18^. People tend to respond to mortality salience by consuming more specific products and services, such as purchasing domestic products and buying brand products^19,20^. They even respond to the negative psychology caused by mortality salience only through consumption, such as increased impulsive consumption^21^, hedonic consumption tendency^22^, and prosocial consumption tendency^16^.

The psychological effects of COVID-19 on consumers have currently been studied from threat perception^23^, price sensitivity^24^, consumption behavior^25^, virtual interaction^26^, and application of artificial intelligence^27^, however, research at the physiological level requires further exploration. Evidence suggests that during the pandemic, the use of artificial intelligence (AI) in virtual travel to provide a physical visual experience can effectively compensate for visitors’ lack of freedom and control, as well as other psychological needs, thereby reducing their feelings of discomfort^28^. Compared to the functional value of the product or service itself, the physical sensory experience induced by sensory consumption, driven by mortality salience, plays a more prominent role in compensating for people’ s psychological needs. Yet, existing studies have not given this aspect sufficient attention. Therefore, it is crucial to investigate sensory compensation issues from a physiological perspective in the study of consumer behavior during the pandemic. This approach can provide a deeper understanding of the relationship between mortality salience and sensory compensation within the context of consumer behavior.

### Sensory compensation

Sensory consumption is a physiological consumption experience that includes one or more of the following senses: taste, sight, hearing, smell, and touch^29^. Sensory consumption is susceptible to psychological state. Consumers prefer strong sensory experiences when emotions are strong. For instance, in the context of Dark Tourism, individuals who are experiencing heightened negative emotional states often use deep dark aesthetics as a medium for pictorial expression^30^. Sensory compensation refers to psychological compensation through high-intensity sensory consumption^31^. Research has demonstrated that a person’s psychological condition will influence their sensory experience. For instance, in front of the same audience, participants in a high-status psychological state reported hearing greater applause compared to subjects who felt they were of lesser social standing^32^. Different levels of sensory compensation will be required as a result of this psychologically induced sensory aberration. According to literature, negative emotional states intensify consumers’ pleasant response to intimate contact, increasing their consumer preference for products that simulate intimate contact^33^. Additionally, sensory compensation can offset unfavorable psychological states using a variety of sensory cues as a cross-domain compensation^30^. When confronted with a threat, consumers tend to gravitate towards high-saturation colors and loud music^7^, seek psychological relief through the consumption of high-sugar foods^27^, or pursue a soft tactile experience^34^. A similar compensatory effect can be achieved by intensely stimulating the consumption of other sensory modalities.

Compared to other senses, there is relatively limited research on taste sensory experiences. However, taste is one of the five primary senses and is particularly sensitive to emotions, as it provides one of the most emotionally charged physical experiences^29,35^. Consequently, taste preferences can vary in response to emotional perceptions on a psychological level. Moreover, taste can compensate for psychological needs by stimulating the taste buds. For instance, when experiencing negative emotions, an individual’ s taste sensitivity may shift, becoming more sensitive to sour flavors while less sensitive to sweetness. This can lead to an increased preference for sweet tastes, resulting in higher consumption of sweet foods as a means of taste compensation to reduce unpleasant emotions^36^. During the pandemic, the negative psychological effects induced by mortality salience may also drive individuals to engage in sensory compensation. It remains to be seen whether this sensory compensation will manifest in the realm of taste and what specific alterations in taste preferences it might bring about.

### Taste and flavor

Taste, as one of the sensory experiences, is an individual’s physical sensation of distinguishing the taste of a substance. Food melts in the mouth, reacting with the tongue, palate, and throat taste buds to cause individuals to sense the flavor^37^. The taste experience process involves millions of chemical interactions, making conclusions challenging. Currently, only five basic tastes—sour, sweet, bitter, salty, and fresh—are understood well^38^. Among these, sodium chloride in food, such as the regular usage of edible salt, is the main source of the salty flavor. Saliva breaks down while being chewed, releasing sodium chloride, which then chemically interacts with taste receptors to produce a salty flavor^39^. A non-basic flavor, spiciness in food is mostly produced by capsaicin, piperine, etc., found in foods like chili, pepper, ginger, etc. These compounds cause acrid, tingling, and burning sensations by stimulating the mucous membranes of the tongue, oral cavity, and nasal cavity. It’s a sophisticated taste perception^40^.

Flavor is the result of taste experiences. People’s taste preferences are not solely determined by individual genes in terms of their formation^41^; they are also significantly influenced by various external factors. These include the availability of food, cultural background, cultural shifts^31^, economic conditions^42^, and major social events^43^, among others. For example, individuals in high-income countries tend to consume more added sugars, South Asians often enjoy richly flavored dishes, and population mobility can alter taste preferences. Additionally, experiences related to emotions such as death and fear, such as those encountered by tourists in black tourism, may lead to a desire for high-calorie hot drinks as a form of compensation^44^. Furthermore, the outcome of a sports event, such as a team losing, can result in fans consuming more saturated fats and increasing their calorie intake^45^. Moreover, psychological conditions can also impact flavor perception^46^. When made to feel grateful, people choose sweets^47^, and most people opt to consume more hot cuisine when they are with violent coworkers^7^. The metaphorical cognition of taste will influence taste choices as well. It’s commonly accepted that spice can increase someone’ s willingness to take risks and engage in conflict^7^. Another reason why people pick foods with strong flavors when they are experiencing negative feelings like stress and oppression is because of this.

During the COVID-19 pandemic, individuals experienced a significant external threat. Terror Management Theory (TMT) posits that such acute external threats elicit existential anxiety, which motivates consumers to seek psychological security^13^, thereby triggering compensatory consumption behaviors^55^. To counteract these psychological threats, compensation can be achieved not only through psychological mechanisms^58^ but alternatively via embodied pathways^87^. Grounded in Embodied Cognition Theory, intense sensory experiences induce heightened somatic satisfaction, which mitigates existential anxiety and insecurity stemming from external threats. Specifically, in terms of taste and diet, stronger flavors can produce a more intense sensory experience^31^. This heightened bodily perception can provide a sense of security and control, thereby alleviating existential anxiety. Collectively, mortality salience may drive consumers to engage in compensatory gustatory consumption through strong-flavored products. Consequently, the following hypothesis was proposed:

#### H1

Consumers who perceive high mortality salience (high-MS) versus low mortality salience (low-MS) would create stronger taste compensation, indicating a preference for foods with strong flavors.

### Compensation motivation

A succession of compensatory acts may also be prompted by compensating motivation in order to lessen the threat’s negative effects^53,54^. Within-domain compensation is the compensation strategy that is directly linked to the danger source^55^. For instance, purchasing books to assist you better your grades following a test failure, purchasing status-related goods when your status is in jeopardy^56^. However, if there are no resources directly related to the threat, people may resort to “across-domain compensation” through indirect approaches^55,57^. For instance, after a team loses a game, fans tend to overindulge in high-fat foods^45^, and people have an addiction to eating sweets^58^. If direct within-domain compensation is impossible, the threat can also be reduced through indirect across-domain compensation. Consumers prefer to engage in non-related activities when faced with threats in order to divert their attention and eliminate the threat^59^. As a result, across-domain compensation can occasionally be more prevalent than within-domain compensation. Research has revealed that people prefer to buy stuffed animals when they’re feeling down in order to get tactile across-domain compensation^33^. The tendency to consume foods with strong flavors after being exposed to mortality salience can be seen as an individual’s across-domain compensation in taste under the pandemic.

#### H2

Compared with low mortality salience (low-MS), stronger compensation motivation will be generated when consumers perceive high mortality salience (high-MS), and furthermore, they prefer to choose a strongly flavored food. That is, compensation motivation has a mediating effect.

### Self-efficacy

People’s tolerance for environmental threats varies due to individual traits, and as a result, they will feel various emotional states. Consumers will have different needs for compensation depending on their unique qualities when faced with mortality salience. As a result, this study uses self-efficacy, or the overall evaluation of one’ s own ability, as a moderator to investigate whether consumers with varying levels of self-efficacy have an inherent compensation mechanism in the face of death threats.

Bandura was the one who initially developed the idea of self-efficacy. According to him, self-efficacy is the conviction that one has the power to carry out a particular conduct or accomplish a particular goal^60^. Then Schwarzer et al^61^ noted that there is a general belief that is reasonably stable, can handle a variety of jobs across numerous fields, and does not alter from field to field, and they also advanced the idea of generic self-efficacy. Self-efficacy influences a person’s motivation, emotions, and even action^62^. Per the studies, an individual’s level of self-efficacy influences their emotional experience; that is, people with high self-efficacy have more confidence, independence, and determination, whereas people with low self-efficacy are more likely to experience depression, anxiety, and helplessness^63,64^. It can also improve people’s cognitive processes, such as consumer perceptions^65^ and decision quality^66^. Furthermore, research indicates that self-efficacy is the primary variable that controls and stimulates human behavior motivation, and it influences people’s behavioral choices in response to threats and challenges^67,68^. Integrating the concepts self-efficacy and motivation regulation: how do self-efficacy beliefs for motivation regulation influence self-regulatory success?^69^ For example, people with high self-efficacy pick more difficult tasks, put more effort into overcoming challenges and achieving goals, and are more tenacious^70^. They also have a higher level of self-confidence in controlling the situation and achieving their goals. Conversely, those with poor self-efficacy put up less effort and perseverance when faced with challenges^71,72^.

Self-efficacy is a crucial personal resource because it is a relatively steady personality attribute^73^. It is essential to support people in their efforts to overcome challenges and deal with risks since belief is one of the fundamental resources required for human existence and development. The strength of people’s compensatory motivation and behavioral intentions in the face of threats will depend on their level of self-efficacy, which is an individual’s subjective and internal resource^74^. Because of this, consumers with high self-efficacy are more self-assured and believe that they can relieve the strain brought on by the COVID-19’s mortality salience through their own efforts without the need for further resource compensation. As a result, compensation motivation will be weakened. While individuals with low self-efficacy will actively seek other resources to compensate for the unsatisfied psychological needs caused by the pandemic’s mortality salience, resulting in stronger compensation motivation. Then the following hypothesis was proposed:

#### H3

**Fig. 1:**
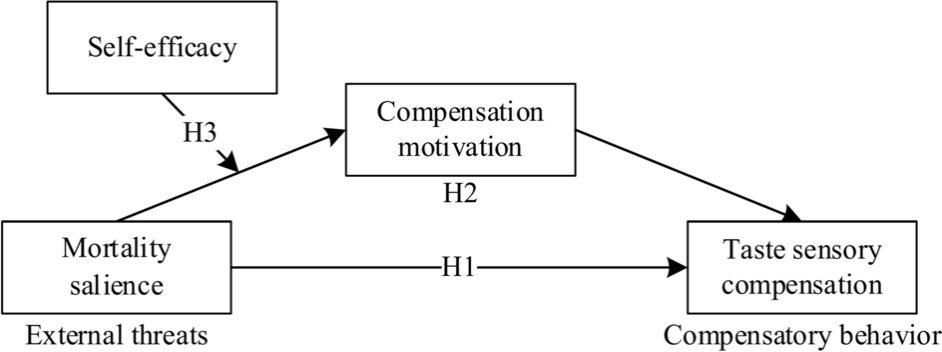
Research model.

The relationship within mortality salience and compensation motivation is negatively moderated by self-efficacy. Specifically, compared to consumers with high self-efficacy, low self-efficacy consumers have a stronger compensation motivation after experiencing mortality salience (Fig. 1).

Summarizing the above research hypotheses into Fig. 1, the article is based on Terror Management Theory and constructs a three-stage framework for external threat psychological compensation motivation and psychological compensation behavior. It demonstrates that mortality salience triggers individual psychological motivation and taste compensation behavior during a pandemic.

### Overview of the studies

To examine how mortality salience influences consumer taste compensation, we conducted a total of six studies, utilizing a combination of secondary data (Study 1) and experimental design (Studies 2a, 2b, 3a, 3b and 4). In Study 1, we examined the correlation between mortality salience and consumer taste compensation during the pandemic using operating data from a real restaurant. Study 2 utilized a situational experiment involving ordering dishes in a restaurant to test the main effect. Study 2a employed a sample of Chinese residents, whereas Study 2b used a sample of foreign students in China. Study 3 involved two field studies to measure participants’ preferences for foods with strong flavors based on their actual taste choices (Study 3a) and consumption (Study 3b), and it tested the mediating effect of compensation motivation. Study 4 used situational simulation experiments to further explore the moderating role of self-efficacy (The data used in this study is available at: 10.6084/m9.figshare.28829762.v1).

## Study 1

### Data

In Study 1, we examined the correlation between mortality salience and consumer taste compensation using real restaurant operating data. The study chose the 2010-founded Laoliehu Restaurant in Chengdu, Sichuan Province, China. It is a famous local Sichuan restaurant. Sichuan cuisine is one of China’s eight major cuisines. It is famous for its spicy seasoning, which corresponds to Western Sichuan’s strong-tasting dietary habits. Laoliehu Restaurant, a well-known local catering company, offers a large selection of classic Sichuan dishes to meet customers’ strong-tasting diet needs, as well as some light-tasting dishes and desserts, considering people of various ages and dietary habits. Therefore, the operating statistics of Laoliehu Restaurant can accurately reflect consumer taste preferences, particularly the predilection for items with strong flavors. It also has a highly consistent customer flow, with an average daily reception volume of about 200, which is helpful for gathering experimental data.

Since April 29, 2020, China’s pandemic spread has been controlled, social order has been restored to most areas, and the tourism sector is slowly returning to normal. Therefore, this study selected the data of the Labor Day break in 2020 (May 1st to 7th, 2020) and the Laoliehu Restaurant receipts data in the same period in 2019 to test the changes in customer tastes under the threat of death caused by COVID-19. The data is provided by the Laoliehu Restaurant, including 73 and 114 receipts in 2019 and 2020, respectively. Each receipt included consumption information about the dishes ordered by guests during their visit. Three chefs coded every dish in every receipt in 2019 and 2020. The specifics are as follows.

### Coding

First, encode the taste of each dish. The coding process was completed by Laoliehu’s executive chef and two vice executive chefs respectively, referring to the *Standards for Preparation of Classical Sichuan Dishes*. Based on the degree of saltiness and spiciness, some dishes such as sweetness, sourness, and bitterness are coded based on sweetness, acidity, and bitterness. The codes have 10 levels, the lowest is 1, which means that the taste is lightest and not spicy at all, such as Sautéed Seasonal Vegetable, Steamed Eggplant with Soya Sauce, etc.; the highest is 10, which means the strongest taste and extremely spicy, such as Boiled Fish with Chili Sauce, Hell Spawn Chicken, etc. The three chefs coded separately, and the intercoder reliability was 0.825. The inconsistent codes were discussed until reached an agreement.

### Results

According to the taste score of each dish, the average score was calculated for all dishes in each receipt (Table 1). Using SPSS v22.0 (IBM Corp., Armonk, NY, USA), a one-way ANOVA was conducted with the average taste score of each dish as the dependent variable and mortality salience (low vs. high) as the independent variable. The results show that compared to 2019, consumer tastes increase significantly in 2020 under the threat of the pandemic (M_low_ = 4.39, SD = 0.976; M_high_ = 4.77, SD = 0.946; F(1,185) = 7.006, *p* = 0.009, η^2^ = 0.036).

**Table 1:** Data descriptive statistics.

	# of receipts	Min	Max	Mean	SD
Taste score in 2019	73	2.00	7.00	4.39	0.976
Taste score in 2020	114	2.00	7.33	4.77	0.946

### Discussion

Study 1 used operational data from a well-known local restaurant to examine the relationship of mortality salience and consumer tastes compensation. It was revealed that consumers’ tastes were stronger during pandemics than that in the pandemic-free period. This proved that customer taste preference was correlated with mortality salience. Study 1 utilized authentic operational data to affirm that the main effect of this study is significant, though it does come with some limitations. The operational data from restaurants might be impacted by other potential factors, such as differences in customer bases. Customer groups may change due to the lengthy interval between the two sets of data, which could potentially impact the study’s results. For this reason, Study 2 further used an experimental approach to reevaluate the major effect and strengthen the findings.

## Study 2

Based on Study 1, Study 2 conducted a simulation experiment with an ordering scenario to evaluate Hypothesis 1 under stricter controls that account for potential interference issues. Study 2a was conducted in China during the COVID-19 pandemic in 2022. To enhance the robustness of the research conclusions, Study 2b was carried out among foreign students studying in China. These students are very familiar with Chinese cuisine, which is advantageous for making accurate choices and avoiding selection bias that could arise from unfamiliarity with the dishes’ flavors.

### Methodology

#### Pretest

This stage tested manipulation materials of mortality salience. The experimental stimulus materials were presented in the form of “COVID-19 briefing news” (Appendix 1). The pandemic was reported positively in low-MS content, and negatively in high-MS material. Referring to previous research^25^, we used two items: “What degree do you think is the risk of infecting COVID-19” and “How much do you scared of the COVID-19” to measure mortality salience perception (7-point scale, 1 = very low, 7 = very high).

All pretest samples were recruited online for a fee. In total, 97 participants (48.5% female; M_age_ = 27.35, SD = 5.743) joined pretest (MS: low vs. high). One-way ANOVA results confirmed that high-MS group participants had a significantly higher perception (*α* = 0.902) than those in the low-MS group (M_low_ = 4.47, SD = 1.434; M_high_ = 5.05, SD = 1.272; F(1,95) = 4.480, *p* = 0.037, η^2^ = 0.045). The manipulations adopted were appropriately designed.

#### Participants and study design

The single-factor between-subject experimental design was adopted in this section (MS: low vs. high). Study 2a collect participants for a paid experiment in the community (57.5% females, M_age_ = 27.58, SD = 6.412). Study 2b collected 198 foreign students studying in China for a fee through the international colleges of Chinese universities (43.4% females, M_age_ = 24.69, SD = 4.048). Participants were assigned to one of these two experimental groups through computer-generated random sequences (in a 1:1 ratio), and the allocation results were saved by independent research assistants in sealed envelopes.

#### Procedure

To manipulate the mortality salience, all participants read a pandemic-related report (Appendix 1), and their perception of mortality salience was measured.

Following that, in Study 2a participants were instructed to pretend a family gathering and to prepare dishes for a ten-person dinner according to the menu. We created a menu based on Laoliehu’s dishes and taste codes (same as Study 1). The menu includes 18 dishes, each with 2–3 degrees of flavor ranging from light to salty and spicy (Appendix 2). The participants were free to select any flavor of each type of food to match the photos and taste ratings on the menu, but each dish could only have one flavor. In the end, a total of 10 dishes were ordered from the participants. Participants finally disclosed their gender and age.

In Study 2b, participants were asked to imagine attending a New Year’s dinner party in China, where each person would choose a small hotpot and select their own hotpot soup base. The menu of the soup base displays 7 different soup bases with varying degrees of spiciness (Appendix 3). In order to more accurately reflect the degree of spiciness of each soup base, the weight of the added spicy seasonings (including chili peppers, chili oil, Sichuan peppercorns, etc.) is marked below each soup base chart. In addition to the seven options mentioned above, experimental participants can fine tune between the two options based on their personal preferences. This quantitative labeling makes taste measurement more accurate.

### Results

#### Manipulation checks

Using SPSS v22.0 (IBM Corp.), mortality salience manipulation was analyzed through one-way ANOVA. In Study 2a, the high-MS group showed significantly higher perception than the low-MS group (*α* = 0.848; M_low_ = 4.67, SD = 1.166; M_high_ = 5.16, SD = 1.232; F(1,137) = 5.638, *p* = 0.019, η^2^ = 0.040). Similarly, in Study 2b, high-MS group participants’ perception was significantly higher than low-MS group participants (*α* = 0.862; M_low_ = 4.14, SD = 1.432; M_high_ = 4.85, SD = 1.416; F(1,196) = 12.026, *p* = 0.001, η^2^ = 0.058). The manipulations adopted were appropriately designed.

#### Dependent variable

**Fig. 2:**
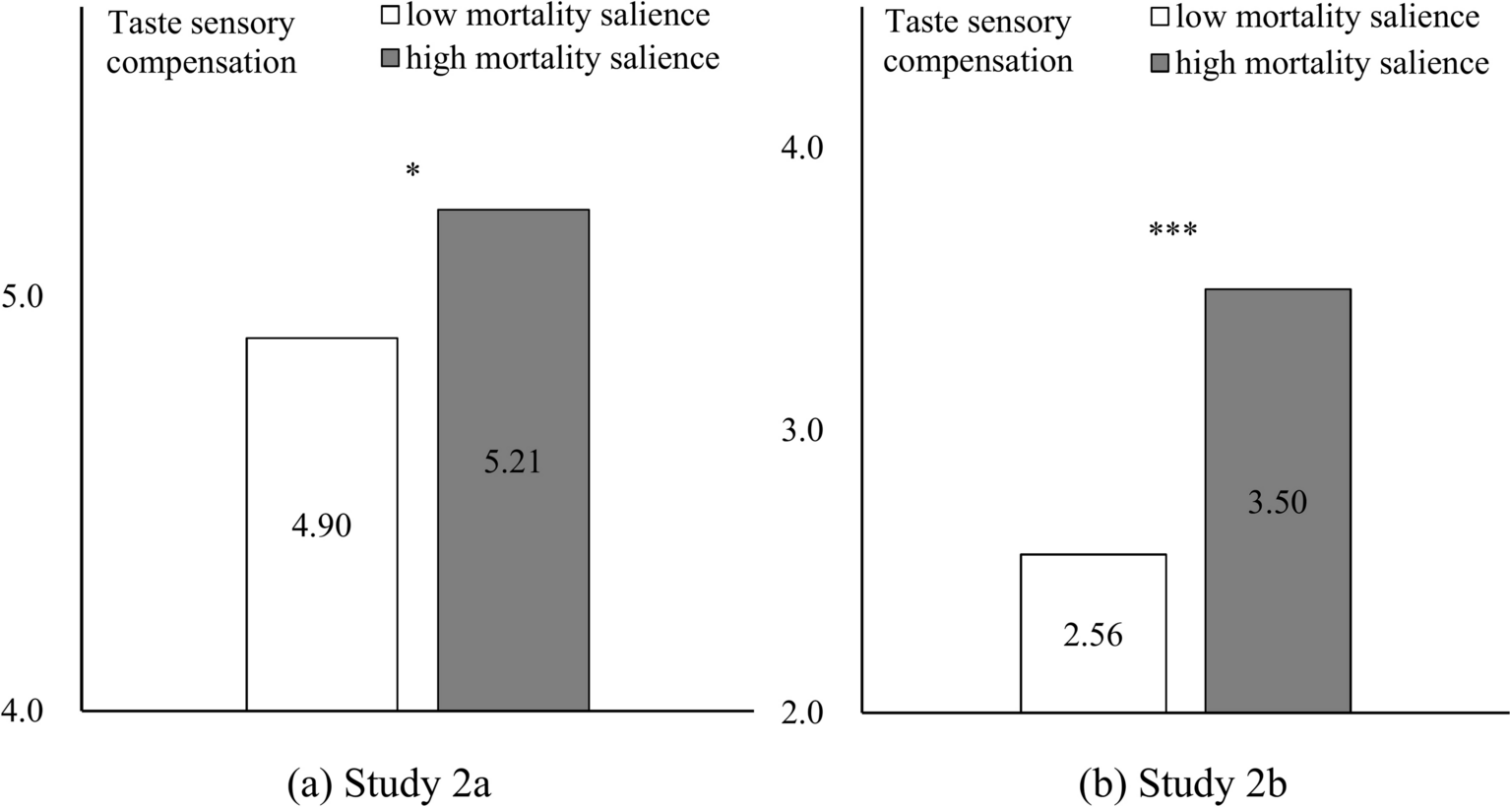
Results of Study 2a and 2b.

Statistical analyses performed with SPSS v22.0 revealed a significant influence of mortality salience on sensory compensation (Fig. 2). Compared to the low-MS perception group, the high-MS perception group selected dishes with heavier flavors, which means a higher taste compensation (In Study 2a: M_low_ = 4.90, SD = 0.875; M_high_ = 5.21, SD = 0.806; F(1,137) = 4.676, *p* = 0.032, η^2^ = 0.033; In Study 2b: M_low_ = 2.56, SD = 1.267; M_high_ = 3.50, SD = 0.937; F(1,196) = 36.013, *p* < 0.001, η^2^ = 0.155). Then, H1 was supported.

### Discussion

Study 2 adopted two ordering scenario simulation experiments, and while controlling for other potentially influencing factors, it has been discovered that people prefer foods with a stronger flavor when faced with high mortality salience as opposed to low mortality salience, demonstrating that mortality salience can impact people’s desires for taste compensation. Two experiments in Study 2 were conducted to test the research hypothesis using two sample groups: Chinese students and international students. The research results demonstrate that the hypothesis is supported across groups with different cultural backgrounds, with consistent taste compensation patterns emerging among both Chinese and international student samples. While cultural dietary preferences manifested in specific flavor choices, these findings collectively indicate that the research conclusions exhibit good robustness across cultural dimensions. Study 2 provided more evidence to re-verify the main effects of mortality salience and taste compensation. Along with Study 1, H1 was supported.

It is important to note that in Study 2, the simulated ordering scenario might not fully encompass the complexity of real-life dining behavior. Consequently, in Study 3, a field study is adopted to test hypotheses using the actual consumption behavior of the experimental participants. There some potential risks in the experiment, such as the spice tolerance, the baseline dietary habits, and cultural taste preferences may affect compensatory behavior of the participants. Thus, in future experiments, it is necessary to control these factors of participants to eliminate potential influence.

## Study 3

Study 3 delves deeper into the psychological mechanisms between mortality salience-and taste compensation, confirming the mediating effect of compensation motivation. In contrast to situational experiment in Study 2, a field study was used in Study 3: two methods were used to measure the strength of taste compensation. Among them, Study 3a provided two flavors of peanuts: spicy and non-spicy. However, Study 3b only provided a popular spicy snack in China called “spicy strips” and measured the participants’ consumption.

### Methodology

#### Participants and design

Both Study 3a and 3b used a one-factor between-group design (MS: low vs. high). We are recruiting participants for a paid experiment within the community. Upon completing the information registration, participants will proceed to our reserved restaurant on the agreed-upon date for the offline field experiments. Totally, 192 subjects participated in Study 3a (56.2% females, M_age_ = 30.31, SD = 5.998) and 179 subjects participated in Study 3b (44.7% females, M_age_ = 29.67, SD = 7.322).

#### Procedure

To ensure the randomness of the experiment, we use the random sequence generated by the computer to achieve the allocation. The subjects were unaware of the group they belonged to. To manipulate mortality salience (MS), subjects were required to read pandemic-related news (same as Study 2), then their perception of mortality salience was assessed. After that, in Study 3a, the subjects were offered a choice between two varieties of peanuts (0 = non-spicy, 1 = spicy; Appendix 4), reported the level of spicy they expect (1 = non-spicy, 7 = very very spicy). Then they were provided the goods they choose. In Study 3b, subjects were able to taste “spicy strips” on site. “Spicy strips” have a spicy taste, allowing the actual consumption to effectively reflect the subject’s preference for foods with strong flavors. Subjects reported the level of spicy they expect (1 = non-spicy, 7 = very very spicy). Before and after the experiment, we weighed the total amount of food and used the difference to calculate the actual eating weight.

Later, all subjects need to give an account of compensation motivation. We used the compensation motivation scale created by Mortimer et al^75^. The six items are: I consume stronger-tasting foods to reduce my stress; I consume stronger-tasting foods to lift myself spirits; I consume stronger-tasting foods to make myself feel better; I consume stronger-tasting foods to make up for a terrible day; I consume stronger-tasting foods to feel relaxed; I consume stronger-tasting foods to please myself (7-point scale; 1 = strongly disagree, 7 = strongly agree). At last, subjects disclosed their gender and age.

### Results

#### Manipulation checks

Using SPSS v22.0 (IBM Corp.), mortality salience was analyzed through one-way ANOVA, participants in the high-MS group exhibited significantly higher mortality salience perceptions than those in the low-MS group (In Study 3a: *α* = 0.823; M_low_ = 4.85, SD = 1.061; M_high_ = 5.24, SD = 0.955; F(1,190) = 6.905, *p* = 0.009, η^2^ = 0.035; In Study 3b: *α* = 0.831; M_low_ = 2.75, SD = 1.028; M_high_ = 4.35, SD = 0.822; F(1,177) = 133.574, *p* < 0.001, η^2^ = 0.430). So, the manipulations were appropriately designed.

#### Dependent variable

**Fig. 3:**
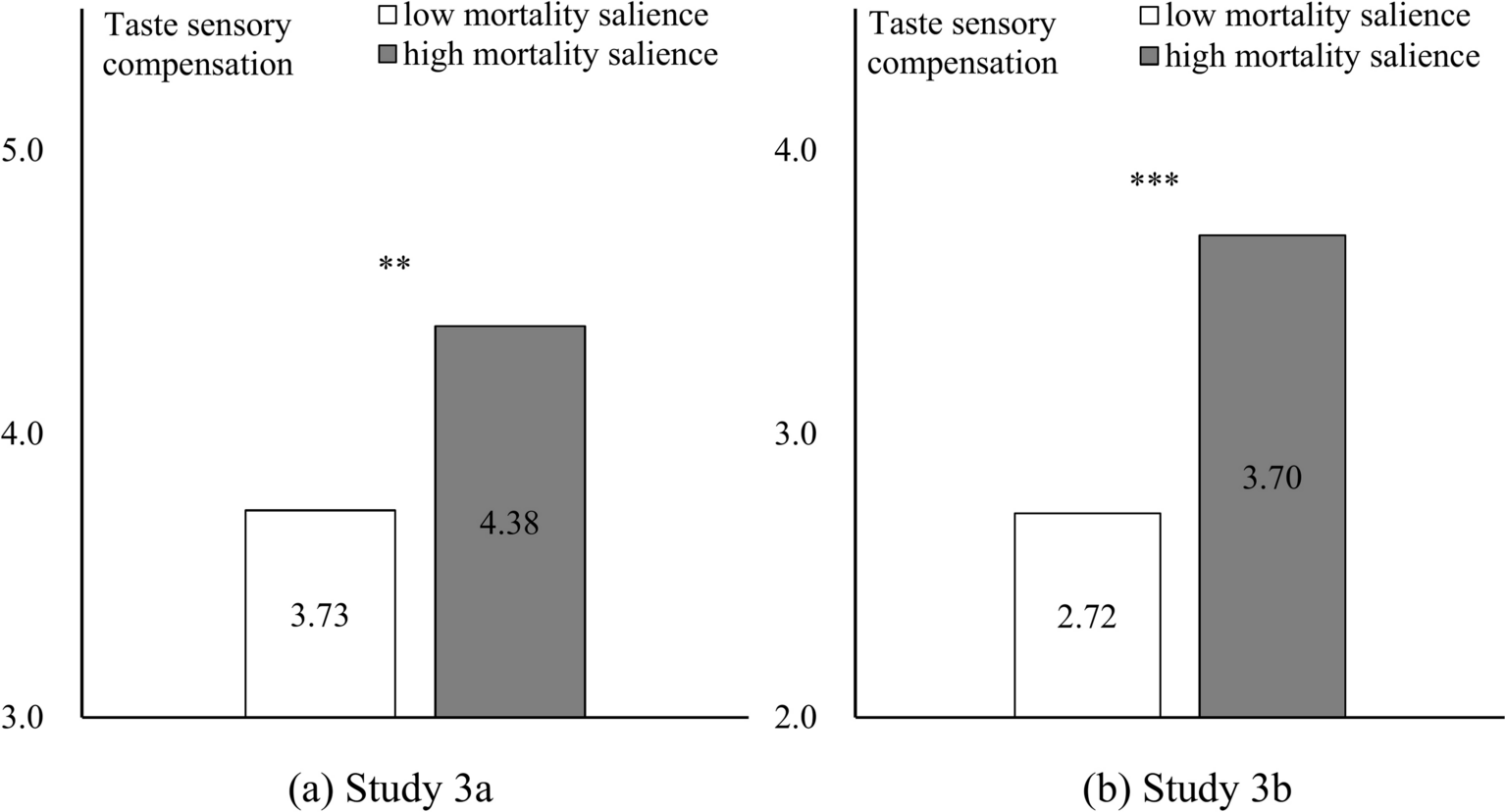
Results of Study 3a and 3b.

SPSS v22.0 (IBM Corp.) was used for analyzing. The results of the one-way ANOVA revealed that (Fig. 3), participants in the high-MS group reported higher level of spicy they expect than the low-MS group (Study 3a: M_low_ = 3.73, SD = 1.704; M_high_ = 4.38, SD = 1.403; F(1,190) = 8.476, *p* = 0.004, η^2^ = 0.043; Study 3b: M_low_ = 2.72, SD = 1.512; M_high_ = 3.70, SD = 1.372; F(1,177) = 20.493, *p* < 0.001, η^2^ = 0.104).

In Study 3a, the Chi-square test results shown the high-MS group more prefer spicy peanuts than the low-MS group does (M_low_ = 0.43, SD = 0.498; M_high_ = 0.63, SD = 0.483; χ^2^(1) = 8.318, *p* = 0.004). In Study 3b, one way ANOVA shows the high-MS group ate more strong taste *Spicy strip* (M_low_ = 66.20, SD = 44.039; M_high_ = 88.01, SD = 37.559; F(1,177) = 12.689, *p* < 0.001, η^2^ = 0.067).

#### Mediating effect

Using SPSS v22.0 (IBM Corp.), compensatory motivation was analyzed through one-way ANOVA. High-MS group exhibits higher compensatory motivation compared to low-MS group (In Study 3a: *α* = 0.903; M_low_ = 5.08, SD = 1.338; M_high_ = 5.64, SD = 1.113; F(1,190) = 9.290, *p* = 0.003, η^2^ = 0.047; In Study 3b: *α* = 0.875; M_low_ = 3.71, SD = 1.430; M_high_ = 4.77, SD = 1.084; F(1,177) = 32.170, *p* < 0.001, η^2^ = 0.154).

The mediating role of compensatory motivation was examined using PROCESS (Model 4). Sample size was set to 5000 bootstrap samples^76^. In Study 3a, the results revealed that age had a significant impact on compensating motive (β = − 0.031, 95% CI = [− 0.061, − 0.001], not included 0), whereas gender had no significant impact on it (β = 0.064, 95% CI = [− 0.302, 0.430], included 0). In terms of taste compensation, gender had a significant impact (β = − 0.746, 95% CI = [− 1.396, − 0.096], not included 0), while age had no significant impact (β = − 0.035, 95% CI = [− 0.089, 0.019], included 0). After controlling gender as well as age, the direct effect indicated non-significant results (direct effect = 0.590, SE = 0.326, 95% CI = [− 0.050, 1.230], included 0), but the indirect effect through compensatory motive was significant (indirect effect = 0.371, SE = 0.158, 95% CI = [0.116, 0.730], not included 0), demonstrating compensation motivation as a complete mediator. Similarly, in Study 3b, the results revealed that the indirect effect through compensatory motive was significant (indirect effect = 25.788, SE = 5.312, 95% CI = [15.624, 36.616], not included 0), the indirect effect through compensatory motive was significant (direct effect = − 5.810, SE = 4.214, 95% CI = [− 14.126, 2.507], included 0). Thus, H2 was supported.

### Discussion

Study 3a and 3b utilized various types of food, employing classification and weight measurement respectively to assess taste preferences. The outcomes of Study 3 indicated that mortality salience correlated with individuals’ compensation motivation and their desire for taste compensation. This finding unveiled the crucial psychological mechanism behind the alterations in consumer word-of-mouth influenced by the pandemic. Study 3a’s limitations include the availability of only two flavors of peanuts and the fact that neither peanuts nor “spicy strips” are typical dishes on restaurant menus, which could affect the external validity of the findings. Consequently, further testing in a more representative dining context is required.

## Study 4

China presently has more than 39,500 hot pot restaurants. In China, hot pot is a widely popular type of cuisine. In order to recreate a hotpot dining experience, Study 4 used a field experiment approach and offered participants a choice of seven different flavors, ranging from clear soup/very light to heavy spicy/very strong. Additionally, differences in individual characteristics must be considered in the impact of mortality salience, so Study 4 examined the moderating effect of self-efficacy.

### Methodology

#### Participants and design

Study 4 employed a 2(MS: low vs. high) × 2(self-efficacy: low vs. high) between-subject experimental design. There were 253 participants (53.8% females, M_age_ = 29.14, SD = 5.451).

#### Procedure

First, 7 flavors (ranging from light to strong) were given to the subjects, and their original taste was assessed using the question “What is your daily diet flavor?” Second, the subjects’ sense of self-efficacy was assessed. The scale was adapted from Schwarzer et al^77^ general self-efficacy scale and consisted of ten items. Same as study 3, a seven points scale was used, where 1 represented strongly disagree and 7 represented strongly agree. Then, the subjects were assigned into the low and high-MS groups randomly generated by the computer, and the mortality salience was manipulated by asking them to read reports about the COVID-19, and their perception of the mortality salience was measured (same as Study 2). After that, the experiment was carried out by simulating the situation where the subjects dining in a hot pot restaurant, with pictures showing a total of 7 different flavors of hot pot seasoning base (from light to strong flavor, Appendix 3) for the subjects to choose from. And the sensory compensation scale about taste was used to measure their compensation motivation (same as Study 3). At last, participants reported their genders and ages.

### Ethics approval

This study received ethical approval from the Ethics Review Committee of the School of Tourism and Hospitality Management at Yunnan University of Finance and Economics (YNUFE). All procedures were conducted in full compliance with government regulations and laboratory’s policies.

1. The principle of voluntariness: the researcher informs the subject or other persons of the possible risks and benefits, and the subject participates voluntarily (by signing an informed consent form). Subjects have the right to withdraw from the experiment at any time during any stage of the experiment without discrimination or retaliation, and their medical treatment and rights will not be affected.

2. Principle of confidentiality: Participation in the experiment and personal data in the experiment are confidential. Only the higher management department and the Ethics Review Committee can access the information of the subjects participating in the experiment according to the regulations.

3. The principle of safety: the concern for the interests of the subjects should be higher than the consideration of scientific and social significance, and the occurrence of serious adverse events in the experiment should be reported to the Ethics Review Committee in a timely manner.

4. Principle of Compensation: In case of damage related to the experiment, the subjects can get treatment and corresponding compensation.

5. The project has no content that jeopardizes national security, involves confidentiality and other contents that are not suitable for public dissemination, and the ideological orientation is correct, and there is no ideological or political problem.

6. Other internationally recognized principles of academic ethical review.

The researchers are requested to carry out the study in strict accordance with the approved research protocol, and any modification should be submitted to the Ethics Review Committee for discussion and approval. Confirmation has been obtained of the informed consent from all subjects and/or their legal guardians.

### Results

#### Manipulation checks

Using SPSS v22.0 (IBM Corp.), mortality salience perception between two groups was significant (M_low_ = 4.50, M_high_ = 4.93, F(1,251) = 5.955, *p* = 0.015) on mortality salience (*α* = 0.813). While for daily taste (M_low_ = 4.12, M_high_ = 4.05, F(1,251) = 0.155, *p* = 0.694) and self-efficacy (M_low_ = 4.27, M_high_ = 4.29, F(1,251) = 0.020, *p* = 0.888), no significant between-group difference was verified. Therefore, the manipulations were appropriately designed.

#### Dependent variable

Results from one-way ANOVA indicated a significantly main effect of mortality salience on taste compensation. The high-MS group had significantly more taste compensation desire than the low-MS group (M_low_ = 4.21, M_high_ = 4.81, F(1,248) = 21.895, *p* < 0.001, η^2^ = 0.076). In this case, H1 was supported again.

#### Mediating effect

High-MS group had higher compensation motivation (*α* = 0.836) versus the low-MS group (M_low_ = 4.22, M_high_ = 5.47, F(1,248) = 95.736, *p* < 0.001, η^2^ = 0.240). According to the mediating effect test method proposed by Hayes (2013), a bootstrap test was adopted to test the mediating role of compensation motivation using PROCESS, Model 4. Sample size was set to 5000 that the same as Study 3^76^. After controlling for three covariate factors (gender, age, and daily taste), results indicated that the indirect impact of the compensating motive was significant (β = 0.159, Boot SE = 0.080, 95% CI = [0.011, 0.329], not included 0). Then H2 was backed.

#### Moderating effect

The moderator role of self-efficacy was tested by the bootstrap approach, using same software and sample size.

**Fig. 4:**
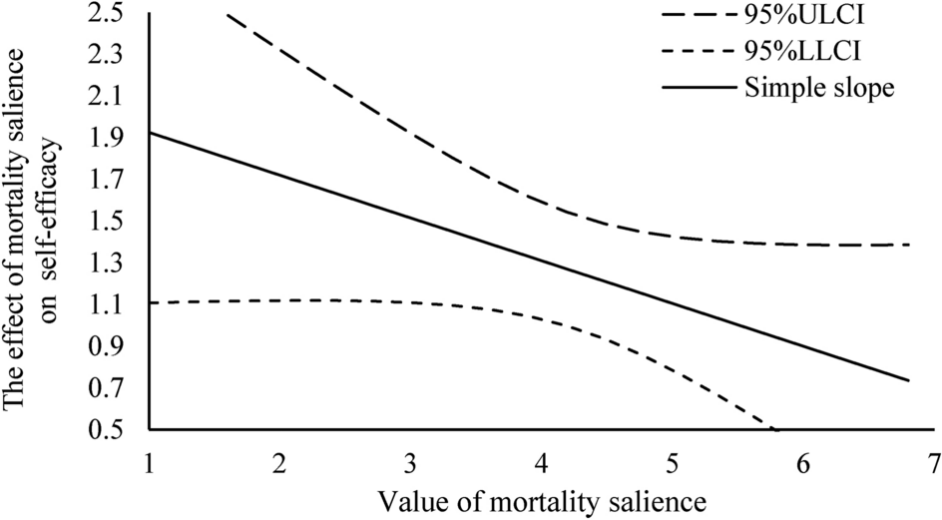
Floot light analysis.

The moderator role of self-efficacy was tested by the bootstrap approach using IBM SPSS Statistics v22.0 (Full name of software and Version number: SPSS v22.0; URL link: https://www.ibm.com/products/spss-statistics). Analytical procedures followed established protocols with the same sample size as above as above, but with Model 7^76^ Outcomes demonstrated a significant interaction effect among mortality salience and self-efficacy upon taste compensation motivation after controlling gender, age, and daily taste (β = − 0.309, Boot SE = 0.115, 95% CI = [− 0.535, − 0.082], not included 0) (Table 2). So far, H3 was supported (Fig. 4).

**Table 2:** Moderated mediating effect of Study 4.

Outcome	Moderator (self-efficacy)	Effect	SE	t	p	95% CI	
						LICI	UICI
Compensation motivation	MS × self-efficacy	− 0.309	0.115	− 2.68	0.008	− 0.535	− 0.082
Taste compensation	MO: − 1 SD, 3.12	0.367	0.108*			0.165*	0.595*
	MO: Mean, 4.28	0.285	0.087*			0.129*	0.470*
	MO: + 1 SD, 5.44	0.202	0.078*			0.076*	0.377*

Note: MS means mortality salience; MO means moderator; * means bootstrap estimation.

When self-efficacy was -1 SD, at the average, and + 1 SD, the mediating impact for taste compensation was 0.367(Boot SE = 0.108, 95% CI = [0.165, 0.595], not included 0), 0.285(Boot SE = 0.087, 95% CI = [0.129, 0.470], not included 0), and 0.202(Boot SE = 0.078, 95% CI = [0.076, 0.377], not included 0) on taste compensation, respectively.

### Discussion

The results of Study 4 confirmed that mortality salience can lead to people seeking stronger taste compensation, as well as the mediating role of compensation motivation. Besides, self-efficacy played a negative moderator role in this process, requiring consumers with low self-efficacy to rely more on external compensation.

## Conclusion

### General discussion

Consumers’ dietary preferences for strong-flavored foods have increased during the COVID-19 outbreak. Based on this phenomenon, this article conducted several experimental studies to investigate the influence of mortality salience upon taste compensation and its mechanism, and the following conclusions are obtained:

First, the mortality salience brought by the pandemic will positively affect consumer’ taste compensation. Mortality salience can affect consumers’ dietary tastes and make them have specific taste preferences. This taste preference is limited in previous studies, such as the sweetness^27^ and high-calorie food^78^. People will also prefer strong flavored diets, such as spicy foods, as noticed during the pandemic. In addition to physical compensation, the strong flavored sensory compensation also has a psychological compensation effect. On the one hand, compared to light-flavor foods, eating foods with strong flavors may better safeguard an individual’s body function and can be physiologically compensated by adding energy. On the other hand, strong stimulation of taste can help individuals cope with psychological threats. When confronted with the COVID-19’s mortality salience, eating strongly flavored foods can provide psychological compensation by stimulating the senses. As a result, in an environment where the pandemic continues to spread, people’s demand for taste compensation will increase, and their dietary behaviors will reflect a preference for items with strong flavors. It should be noted that this change in preference is not only during the COVID-19 pandemic, but also when people feel similar external threats again.

Second, taste compensation, which is affected by mortality salience, is a type of across-domain compensation. Mortality salience is psychological threat perception, but the need for taste compensation arising from exposure to mortality salience are on the physiological level and not directly related to the mortality salience. Taste compensation is across-domain compensation behavior that is triggered by mortality salience in the pandemic and is compensated by physiological compensation psychology. Usually, both within-domain compensation and across-domain compensation can compensate for the psychological needs of individuals. However, compared with across-domain compensation, within-domain compensation will increase the attention to threat information^55,79^, which in turn will enhance threat perception and weaken the effect of psychological compensation. Therefore, when encounter with vital mortality salience brought by the COVID-19, people tend to make across-domain compensation, such as eating strong flavored food for taste compensation, to reduce the personal perception of death threats.

Third, compensation motivation plays a mediating role between mortality salience and taste compensation. Compensation motivation mainly stems from the dissatisfaction with the individual’s basic psychological needs. It will encourage people to seek other resources for physiological compensation^80^. According to Terror Management Theory, during the pandemic, mortality salience acts as a psychological threat, triggering dissatisfaction with various psychological needs such as security and control. This, in turn, activates compensation motivation. Furthermore, compensation motivation drives individuals to actively seek resources through various means to fulfill their psychological needs. Experiments have confirmed that under pandemic conditions, compensation motivation prompts individuals to seek physical sensory satisfaction to address their psychological needs. This is a process where psychological threats lead to physical compensation. As an internal driving force, compensation motivation encourages individuals to pursue taste compensation.

Fourth, self-efficacy can moderate the influence of mortality salience upon consumer compensation motivation. Self-efficacy, as an internal individual resource, can provide belief support for people to deal with mortality salience. When the self-efficacy level is high, people’s motivation to seek external resource compensation is weakened, and when the self-efficacy level is low, this motivation is stronger. Based on previous studies, self-efficacy, as an internal resource, plays an important role not only in motivating behavior^81^, maintaining emotional well-being^82^, etc., but also in helping people resist the psychological threat from mortality salience. We have confirmed through empirical research that self-efficacy has a negative moderating effect on the impact of mortality salience upon consumers’ compensation motivation.

### Theoretical contributions

This research has the following four theoretical contributions:

First, this study discovered the changes in external environmental threats to customers’ catering tastes and found new antecedents of catering tastes. We found that under threat of the pandemic, consumers prefer strong flavored foods. This means that customers’ tastes are not only consciously influenced by factors such as health philosophy^83^, food labeling^84^, advertising^85^, etc., but also unconsciously affected by the external environment. This provides a new idea for the exploration of the influencing factors of customers’ food and beverage taste in the future, Researchers should not only focus on factors that directly influence taste consumption but also investigate the indirect factors affecting taste consumption from the perspective of taste psychological compensation. This includes the psychological and taste compensation needs that arise from external threats such as diseases, natural disasters, and economic downturns.

Second, this study expanded the research scope on pandemic influence. Current research on the COVID-19 mainly focuses on the psychological level of consumers and their consumption behavior^23,86^. However, we discovered that, in addition to psychological and behavioral aspects, the pandemic’s mortality salience will unconsciously enhance consumers’ action in physiological taste compensation. Based on cross-modal correspondence and sensory compensation in the past^87,88^, this conclusion extends that relationship between mortality salience and taste compensation and further explores taste’s across-domain compensation behavior in the sensory field.

Third, based on the principle of compensatory consumption, this study links psychology and physiology to explore the effects of pandemics on consumer tastes and provides a new perspective on the mind–body connection for the COVID-19 study. Currently, most research examines the pandemic’s effects from a single psychological or physiological standpoint^17,89^. However, there is a strong link between mental state and physical experience^29^, and occasionally consumers’ mental states unintentionally have a direct impact on their physical experiences^36,80^. Thus, this study integrates psychological and physiological viewpoints, focusing on the physiological compensation processes induced by psychological demands, to investigate the effects and mechanisms of mortality salience on taste compensation. It aims to complement ongoing pandemic research. The article uncovers the intricate interplay between mortality salience, sensory compensation, and consumer behavior within restaurant settings.

Fourth, this study demonstrates the inherent process of taste compensation for mortality salience. Currently, the intrinsic process of how negative psychological states, such as anxiety, tension, melancholy, etc., affect taste preferences is still unclear^27,90^. We analyze changes in consumer taste preferences during the pandemic from the perspective of sensory compensation, revealing the mediating role of compensation motivation in the impact of mortality salience on taste compensation and adding to the literature on the mechanism of psychological state and taste preferences. Furthermore, this study introduces self-efficacy as a moderator, examining differences in compensation motivation among individuals when faced with mortality salience. Building upon the theory of resource preservation, this further elucidates the mechanism of mortality salience on taste compensation from the perspective of dynamic balance. In other words, if an individual’s internal psychological resources are inadequate to cope with the mortality salience induced by the external environment, they will proactively seek alternative resources to compensate. This enables them to address threats and sustain equilibrium between internal and external environments.

### Managerial implications

Although this study was undertaken in the midst of the pandemic, its relevance is not just for the present. Consumers may continue to perceive the COVID-19 as a threat even after the epidemic has ended, changing their taste preference. Moreover, other global crises, such as pandemics, natural disasters, or economic downturns, also heighten mortality salience. Consequently, the conclusions of this study hold broader management significance for the operation of the hospitality industry, both during the epidemic and in the global crises following COVID-19.

Firstly, during the pandemic, the F&B departments of hotels and restaurants should introduce dishes with more robust flavors. Specifically, to meet evolving consumer preferences, they should prioritize innovation in product formulation. This involves introducing spicier, bolder-flavored dishes with heightened pungency, while gradually phasing out milder options. Building on this foundation, menus should be redesigned to prioritize strong-flavored dishes as front-page recommendations, while launching marketing campaigns to promote limited-time offers on these bold-flavored specialties. This strategy directly addresses consumers’ taste compensation needs by amplifying flavor intensity and visibility. Beyond hotels and restaurants, other food-related industry sectors should promptly adjust their product flavors or introduce new products with more robust tastes to meet customers’ taste demands. Specifically, fine dining establishments could experiment with dishes featuring stimulating premium spices such as Wasabi, Clove, and Sichuan Pepper. The fast food restaurants could offer spicy dipping sauce packets during the pandemic—such as McDonald’s Hot Sauce and Buffalo Sauce packets, or Subway’s Jalapeño Sauce packets—would align with this strategy. The food retails can not only provide more bold-flavored seasonings and spices but also introduce more spicy food items and display them on high-traffic store shelves.

Secondly, consumers affected by the pandemic are motivated to seek compensation. To capture customers’ attention, the hotel’s F&B department and restaurants are introducing and marketing unique, strongly flavorful dishes. They are promoting these robustly flavored dishes to satisfy consumers’ desire for compensation, thereby effectively attracting them. In other words, during the marketing and promotion process, it’s not just about highlighting the taste of the dishes but also about addressing the need to compensate external threats. These strongly flavored dishes provide sensory satisfaction to consumers, offering them a sense of psychological safety. This compensation strategy proves to be particularly effective in drawing in consumers.

Thirdly, hotels and restaurants should identify the pandemic’s node and make timely adjustments to their culinary offerings. Given that the pandemic’s effects are enduring and evolving, and considering that different regions are at various stages of the pandemic, people’s perception of the threat changes over time and location. For instance, China has surpassed the pandemic’s peak, while the United States, Italy, Spain, and other nations are still experiencing its spread^91^. Consequently, consumers’ taste preferences are shifting dynamically due to the pandemic, necessitating those hotels and restaurants adapt their menus accordingly. For example, they should be ready to shift from lighter to richer flavors as the pandemic intensifies. Foods should be saltier and spicier during the pandemic’s growth stage and until it reaches its peak. Menus would gradually revert to a more balanced taste profile post-pandemic.

Fourthly, the hospitality industry requires long-term strategies for post-pandemic recovery. Although the COVID-19 epidemic has subsided, today’s society continues to face the threat of various infectious diseases. For instance, winter is a peak period for miscarriages, accompanied by a surge in news coverage. This intensifies the perceived threats during this season. Consequently, restaurants should also be mindful of the psychological threats faced by customers and adjust the flavor profiles of their food to align with evolving preferences. The dynamic management of these flavors should become a long-term business strategy for the hospitality industry.

### Limitations and future research

This research has some limitations that necessitate further investigation. Firstly, the study primarily employs experimental methods, and the sample size and representativeness are clearly limited. To enhance the credibility of the findings, future research could collect more operational data from larger meal delivery platforms such as Meituan and Elema, and conduct studies with a larger sample size and consumer preferences for receipt-based data analysis. Secondly, the study focuses on how the pandemic has affected taste in the Chinese context and uses only Chinese inhabitants as samples. To confirm the applicability of the findings, future research could include more nations or regions, such as Asia, the Americas, and possibly Europe. Thirdly, while multiple methods were used to measure taste in this article, more precise tools are needed for accurate measurement. The use of sensory panels or biometrics to enhance reliability in future research is considered. Fourthly, potential biases introduced by hypothetical scenario studies (e.g., ordering tasks), potential demand characteristics, and generalizability issues due to cultural sampling (Chinese and international students in China) should be taken into consideration. In future experiments, these potential factors can be controlled and the possibility of them being used as moderating variables can be considered. Fifthly, the current evidence only supports the sense of taste empirically. Future studies could explore how consumers compensate for their other senses, including vision, hearing, smell, touch, and others, in the face of public health crises such as the pandemic, influenza, or other contagious diseases. Previous studies have found that an increase in psychological stress can lead to a need for warm tactile sensations^44^. Future research could further examine whether the threat of COVID-19 increases people’s willingness to seek warm tactile experiences. Sixthly, our findings capture immediate behavioral responses, the temporal persistence of crisis-induced taste changes need further exploration. Longitudinal data is needed to determine whether these altered consumption patterns represent transient coping mechanisms or enduring preference transformations. Recent consumer research suggests that crisis duration and recurrence frequency critically shape habit formation processes^92^. Subsequent investigations could employ multi-wave panel tracking the preferences of participants under threat and after threat disappears, in order to understand crisis-induced taste changes persist over time.

## Supplementary Information

Supplementary Information.

## Data Availability

The datasets used and analyzed during the current study are provided within the supplementary information files. For further data access or correspondence, please refer to the link: 10.6084/m9.figshare.28829762.v1.
