# Supplementary material for: The threat of COVID-19 will influence consumers’ taste compensation
Source: Sci Rep. 2025 May 30;15:19052. doi: 10.1038/s41598-025-01245-2 (PMC12125203; doi:10.1038/s41598-025-01245-2)
Supplement: Supplementary file 1 — Supplementary Information. [file 41598_2025_1245_MOESM1_ESM.docx]

**Appendix**

**Appendix 1**. Reports about pandemic in Study 2

*Low mortality salience.*

The COVID-19 is generally susceptible to the population, but the contagion is controllable. The number of new positive cases in our country have almost disappeared. Compared with the SARS virus, the COVID-19 has the characteristics of mild infection and complete cure. At present, the confirmed and suspected patients have been controlled, and those with latent infection and asymptomatic infection who have no obvious symptoms have gradually realized pre-monitoring. With the gradual restoration of going back to normal life, the risk of a rebound caused by the increase in the flow of people and gathering will still be under control for a long time. Considering the continued spread of overseas pandemic, imported cases have been under control nationally and will not trigger a second outbreak of local pandemic. At present, medical research on the virus is gradually deepening, and specific drugs and vaccines are already in the development stage. If the research and development progress smoothly, the vaccines and specific drugs under development will be available at the end of the year, and the end of the pandemic is just around the corner. After the successful development of the vaccine, it is only a matter of time before the large-scale vaccination is achieved. Therefore, the country is expected to end the current epidemic risk as soon as possible.

*High mortality salience.*

The whole population is susceptible and highly contagious by the COVID-19. During the peak period, the number of new positive cases in the country was as high as 2,000. Compared with the SARS virus, the COVID-19 has the characteristics of rapid spread and difficult prevention and control. At present, the confirmed and suspected patients have been controlled, but the incubation period and asymptomatic infections who have no obvious symptoms have not been fully monitored. As the order of life go back to normal gradually, the risk of a rebound of the pandemic caused by the increase in personnel flows and gatherings still exists for a long time. In particular, the spread of the overseas epidemic has accelerated, and imported cases have become the main body of new cases in our country, which may trigger a second outbreak of the local pandemic. At present, medical understanding of the virus is still limited, and specific drugs and vaccines are still in the development stage. If the virus mutates suddenly, the vaccines and existing therapies under development are very likely to fail, and the development of the epidemic is still unknown. After the vaccine is successfully developed, it will take a certain period to achieve large-scale vaccination, so the country will still face severe pandemic risks for a long time.

**Appendix 2**. The menu used to simulate a la carte in Study 2

**Menu**

**Vegetables:**


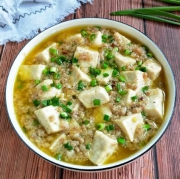


01.

Clear-oil tofu


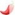


02.


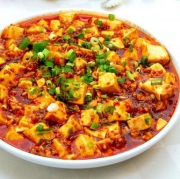


Mapo tofu


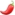

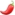

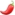

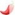


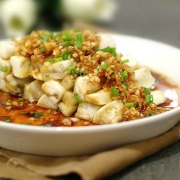


White eggplant


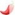


03.


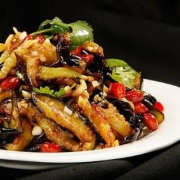


Spicy eggplant


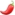

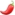

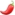


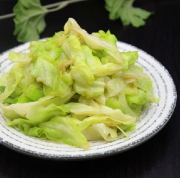


Stir-fried seasonal vegetables


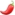


04.


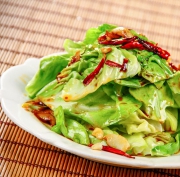


Spicy seasonal vegetables


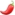

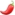

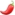


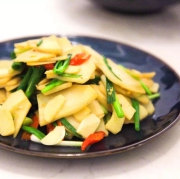


Stir-fried bamboo shoots


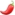


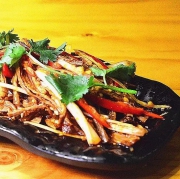


Spicy shredded bamboo shoots


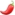

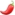

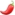


05.


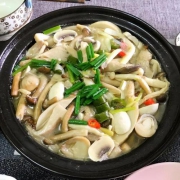


Stewed mushroom


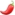

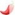


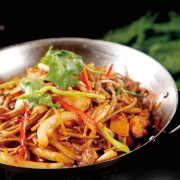


Spicy mushroom


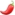

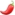

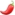


**Fish：**

06.


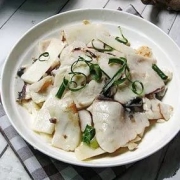


Stir-fired fish filets


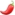

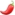


07.


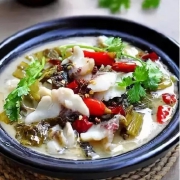


Boiled fish with picked cabbage and chill


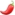

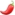

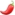


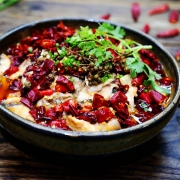


Fish filets in hot chili oil


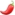

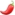

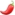

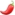

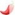


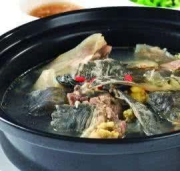


Turtle Soup


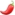


08.


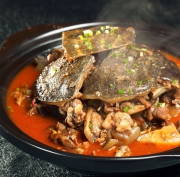


Secret recipe turtle


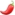

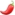

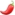

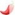


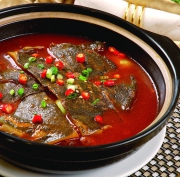


Braised turtle in Brown Sauce


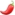

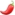

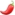

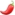

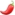


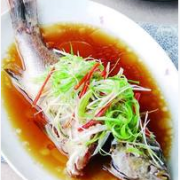


Steamed fish


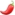


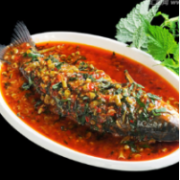


Braised fish with agastache rugosa


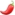

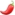

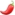

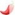


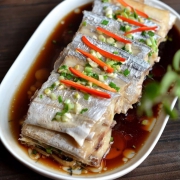


09.

Steamed hairtail


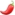


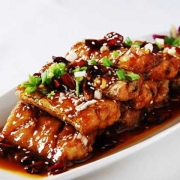


Braised hairtail in brown sauce


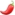

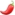

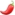

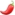

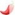


**Meat：**

10.


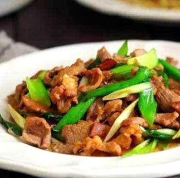


Stir-fried streaky pork with Scallion


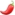

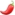

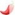


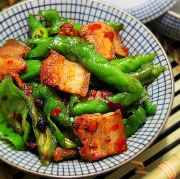


Stir-fried twice-cooked pork with Pepper


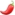

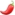

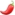

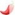


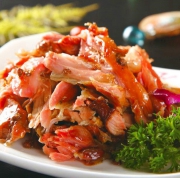


11.

Spiced rabbit meat


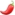

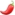

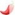


12.


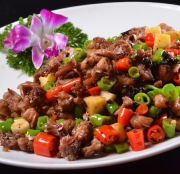


Stir-fried rabbit with ginger


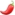

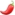

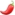

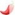


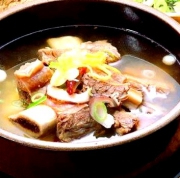


Spareribs soup


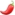


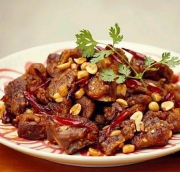


Spicy spareribs


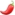

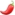

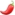

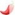


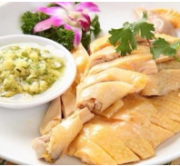


13.

Sliced boiled chicken


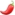


Griddle cooked chicken with Pepper

14.

Stewed duck with mushroom

15.

Griddle cooked duck with Pepper

Sliced boiled beef

Sauced beef

Beef filets in hot chili oil

**Seafood:**

16.

Steamed shrimp with garlic

17.

White shrimp in Typhoon Shelter

Spicy shrimp

Crab with vermicelli

18.

Spicy crab

Rice with abalone

Abalone with Chinese prickly ash

**Appendix 3.** Pictures for choosing the flavor of the hot pot seasoning base in Study 4

**Appendix 4.** Picture for choosing peanut flavor in Study 3

Non-spicy Peanuts Spicy Peanuts
